# For Whom Money Matters Less: Social Connectedness as a Resilience Resource in the UK

**DOI:** 10.1007/s11205-014-0858-5

**Published:** 2015-01-06

**Authors:** Lindsay Richards

**Affiliations:** Cathie Marsh Institute for Social Research, University of Manchester, 2.12A, Humanities Bridgeford Street, Manchester, M13 9PL UK

**Keywords:** Life satisfaction, Money happiness, Income, Connectedness, Social capital, Resilience

## Abstract

The current literature shows that both absolute and relative income are important for happiness, but there is little work emphasising how the strength of the relationship is dependent on personal and social factors. I hypothesise that social connectedness influences the money-happiness relationship because the effect of money is in part felt through the acquisition of social status, whereas status (and associated psychological benefits such as self-worth) can alternatively be gained through social connectedness. In particular, it is theorised that ‘weak ties’ when co-existing with good quality informal ties have a separable and additional benefit to subjective well-being, and that it is the socially isolated who have the most to gain from doing well financially. Social connectedness is conceptualised as a ‘resilience resource’ which has a buffering effect on subjective well-being. Data from the British Household Panel Survey are used, first to establish a measurement schema of connectedness using latent class analysis, and secondly in a multilevel model of life satisfaction with observations from seven consecutive years nested within individuals. The results show that connectedness makes a larger difference to satisfaction in times of financial stress, and that the satisfaction of the socially isolated can ‘catch up’ to some degree with those with larger networks when things are going well financially. The findings also confirm that those for whom money makes the smallest contribution to happiness are those with both strong and weak ties. Weak ties have an additional benefit compared to having strong ties alone. In summary, connectedness has the power to narrow the well-being gap that exists between times of financial struggle and times of relative comfort. It suggests that the psychological benefits of social integration have the capacity to displace money as a source of status and self-worth, and similarly that the importance of money may be exaggerated where these psychological gains are not available through other domains of life.

**Electronic supplementary material:**

The online version of this article (doi:10.1007/s11205-014-0858-5) contains supplementary material, which is available to authorized users.

## Introduction and Background

Does money make us happy? There is plenty of evidence that it does. There is a positive association between subjective well-being (SWB) and GDP at the country level and within countries there is a positive relationship between the income and happiness of individuals (Veenhoven 1993; Diener et al. 1993). Traditional economics has it that the underlying mechanism is direct and causal in that money enables individuals to spend and consume in ways that contribute to their well-being. The list of items desired in life and made accessible with money includes material goods, pleasurable experiences, better health, and better security and safety (Lucas and Schimmack 2009). Health research too offers an explanation based on the direct and causal benefits of money namely that psychological stress is caused by living with an income level insufficient to manage needs (Adler and Snibbe 2003).

On the other hand, psychology provides accounts of the money-happiness relationship that go beyond ‘buying power’. Adaptation theory, for example, describes the process of reversion to some baseline level of happiness following temporary highs or lows brought about by life events such as winning the lottery (Brickman et al. 1978) or receiving a pay-cut (Wolbring 2011). The baseline or “set-point” level of happiness may be largely determined by genes (Tellegen et al. 1988) and corresponds to personality traits such as neuroticism and extraversion which are assumed to be stable in adulthood (Costa et al. 1987; Mccrae and Costa 1991). These traits in turn also influence the coping processes that facilitate reversion to baseline after a negative event (Mayer and Faber 2010).

In addition to the comparison with ‘former selves’ that occurs in adaptation processes, social comparison also occurs, where the point of contrast is other people. The relevant group for comparison (‘the reference category’) is often assumed to be “people like me” where the likeness is determined on salient factors such as age group, educational level and occupation type (Clark and Oswald 1996). When the average income of the reference group is included in regression models alongside personal income, the coefficients tend to be ‘equal and opposite’ (Clark et al. 2008); that is, higher income in one’s reference group will pull down, and even cancel out, any positive effect of personal income. Comparison processes emphasise the importance of *relative* rather than *absolute* income and are used to explain the ‘Easterlin paradox’ whereby average life satisfaction has not improved through time despite large increases in average real income (Easterlin 1995; Clark et al. 2008; Layard 2010). Social comparison is directed either ‘upward’ towards those with higher incomes, better jobs and so on, resulting in lower happiness; or ‘downward’ towards those worse off with a resulting appreciation for what we have and improved well-being (Wills 1981; Taylor and Lobel 1989; Wolbring 2011).

In fact neither the traditional economic explanation, nor comparison and adaptation theories are able to entirely explain the money-happiness relationship. The buying power account is backed to some degree by evidence of a threshold above which happiness gains level off and by the fact that progressively smaller returns are gained as we go up the income scale (Layard et al. 2008). The threshold effect is more evident where the outcome of the study is positive affect (Kahneman and Deaton 2010). However, where the outcome is life satisfaction, the relationship with the log of income is linear (Stevenson and Wolfers 2008) and those earning in the highest income bracket are happier than those in the second highest, who are happier than those in the third, and so on (Lucas and Schimmack 2009).

The evidence for an influence of social comparison is strong but empirical work has demonstrated that the process is not ‘automatic’, which is to say that it is not inevitable or experienced in the same way across individuals or situations. Firstly, reference groups may be actively chosen (Falk and Knell 2004) and downwards comparison is used as an ‘active and cognitive’ coping mechanism (Wood et al. 1985); and secondly, happy people appear to engage in less comparison altogether (Clark and Senik 2010). It suggests that individual and situational differences may be able to explain the degree of comparison undertaken and/or its effect on happiness.

Another area commonly neglected in the literature is the idea that gains in happiness from money reflect the higher rank in society that money brings (Layard 1981). The importance of status for happiness is particularly salient where survey methods are used to acquire measurements because the question “… [draws] people’s attention to their relative standing in the distribution of material well-being and other circumstances” and therefore “… [evokes] a reflection on relative status” (Kahneman et al. 2006, pp. 1908–1909). It follows that other means of achieving status, with the associated psychological benefits such as self-worth and perceived control, may lessen the extent to which money can influence happiness.

This research proposes that social connectedness is such another means. Social interactions, as experienced by the individual, shape the cognitive processes that bring about SWB. Connectedness has the ability to enhance self-esteem (Brown and Harris 1978), provide purpose and meaning (Thoits 2013), self-worth (Weiss 1974) and a sense of control (Johnson and Krueger 2006) and so has the potential to intervene in the money-happiness relationship. The outcomes of this study contribute to a growing literature on mediating factors in the money-happiness relationship including personal values and aspirations (Kasser and Ryan 1993; Nickerson et al. 2003), religion (Lelkes 2006), and political orientation (Di Tella and MacCulloch 1999).

I begin by outlining the concepts of SWB and resilience that are central to the research. This is followed by an overview of social networks and well-being and the specific ways that connectedness might be expected to influence the money-happiness relationship. The empirical analysis is in two parts; the first to establish a measurement schema of connectedness and the second to explore resilience by connectedness. A discussion and conclusions then follow.

### Conceptualising Well-Being

Well-being, broadly speaking, can be dichotomised into the objective and the subjective, where objective well-being may be assessed through health, the meeting of material needs and so on. It is SWB that is central here, and this research focuses on the variation across individuals in the extent to which one aspect of objective well-being (money) influences the subjective. “Subjective well-being” (SWB), too, is an umbrella term which covers the three dimensions of positive affect, negative affect, and life satisfaction (Gasper 2010). The outcome of particular interest in this study is life satisfaction, a question asked in surveys that demands of its respondents the act of cognitive evaluation and appraisal of life circumstances to determine a position on a scale. In choosing this conceptualisation of SWB, priority is being given to individual judgements of meaning and emphasises reflectiveness and meaningfulness above pleasure (ibid). Throughout the sections that follow *SWB* is used synonymously with *happiness* and in doing so follows much of the literature. The phrase ‘money-happiness relationship’ is used as shorthand to cover the effect of income, perceived income, material well-being and so forth on SWB.

### Resilience

This study borrows from developmental psychology (and the natural sciences before) the term *resilience* because of its semantic usefulness in capturing both the outcome and the stressor in a single term. The literature overlaps considerably with some of the key concepts for understanding SWB and has a shared vocabulary including *social support, coping,* and *adaptation*. Resilience is commonly considered a trait of the individual or community under scrutiny but the definition applied here follows many researchers in the field who reject the ‘trait model’ in favour of the ‘process model’. The process model offers a conceptual advantage by encouraging attention to the processes of managing, coping, adapting, maintaining (Masten and Wright 2010) but also to the dependence on context and resources (Cicchetti and Garmezy 1993). In the trait model, resilience may be seen as a parallel construct of, for example, extraversion, which is linked to various positive outcomes. The process model, on the other hand, considers these traits to be ‘protective factors’ or ‘resilience resources’ and are characteristics that make resilience more likely. A resilience approach reveals not just the correlates of SWB, but instead emphasises inequality in the stress experience. In doing so it recognises the role of resilience resources in people’s lives as a way of maintaining or buffering SWB in the presence of stressors.

This research is concerned with the role of connectedness as a protective factor where the stressor under investigation is current financial situation. Resilience is conceptualised as a better outcome than might be expected under the circumstances. In this it is essentially relative in nature and determined by comparing the outcomes of one group to another.

### Social Networks and the Money Happiness Relationship

Social networks have a fundamental role in the maintenance of well-being both ‘subjective’ (Kahneman et al. 2006; Diener et al. 1999) and ‘objective’, e.g. health (Berkman and Syme 1979; Cacioppo and Patrick 2008). Indeed, close supportive relationships are considered a ‘necessary condition’ for SWB (Diener and Biswas-Diener 2008). Below, the mechanisms by which connectedness influences the money-happiness relationship are described concentrating on the following pathways; resource-based benefits, social support, other psychological benefits, and values.

#### Resource-Based Benefits

To the extent that happiness gained from money results from the consumption of goods and services acquired with it, social ties can also be expected to exert a direct influence by providing access to resources. The social capital literature provides evidence that social ties can have direct economic benefits; wide social circles with plenty of ‘weak ties’, for example, can provide access to improved opportunities in the labour market (Granovetter 1973). Access to informal help, such as child-minding and short-term loans or other practical help (Wellman and Wortley 1990) has real financial benefits, and therefore can compensate for low income or high costs, and contributes to SWB in the direct and causal manner assumed in traditional economics.

#### Social Support

In addition to access to employment, loans and practical help which are fungible with economic capital, social ties provide access to emotional and informational aspects of support (Thoits 2011). Social support provides a “protective shell” during times of stress (Holmes 2002, p. 81) and has been shown to buffer the adverse effects of stressful life events on well-being. Economic or non-economic, social capital and social support theories have in common the core notion of resources, for which social ties serve as a conduit. These resources then compensate for, buffer the negative effects of, or provide information that aids avoidance of, financial stress. Financial stress follows ‘losses’, for example, through pay cuts or a reduction in employment hours, or by the lifestyle losses incurred by rising costs. “Loss aversion” theory (Tversky and Kahneman 1991) posits that the effect of a loss is far larger than the opposite consequence of experiencing a commensurate gain, and this has been demonstrated empirically with income (Wolbring 2011).

#### Psychological Benefits

It is not only the valuation of life situation but also self-evaluation more broadly that occurs through social comparison and reflection (Festinger 1954; Thoits 2013). It follows that connectedness has the power to influence self-worth and self-esteem which in turn are associated with happiness (Lewinsohn 1991). Role identity theory posits that purpose and meaning are gained from the normative behavioural expectations evident in different social settings accessed through different roles (Moen 1989).

#### Pro-Social Goals

The direct and causal link between money and happiness is loosened by the weak correlation between *actual* financial resources and *perceptions* of the financial situation (Johnson and Krueger 2006). Aspirations and goals, along with adaptation, influence this relationship between the actual and the perceived. If the gap between actual income and aspired income is large then the subjective appraisals of life satisfaction are negatively affected (Michalos 1985). Goals and aspirations in turn are influenced by values, one of the functions of which, according to Rokeach (1973), is as a standard against which beliefs and actions are justified, which in turn enable the maintenance and enhancement of self-esteem.

Those who prioritise material goals above social ones experience more depression and anxiety (Kasser and Ryan 1993) as well as lower life satisfaction (Headey et al. 2010) and those prioritising money as young adults will go on to report lower satisfaction with friendships later in life (Nickerson et al. 2003). Work examining the effect of early life experiences finds that the prioritisation of financial success over pro-social goals is more common among young adults with less nurturant parents (Kasser et al. 1995). In short, where priority is given to material goals, less emphasis is placed on the social, and more isolated people are likely to care more about money.

### The Role of Weak and Strong Ties

There is plentiful evidence showing that informal strong ties are the ones that provide emotional support, love and sympathy, as well as providing practical and financial help (Wellman and Wortley 1990; Thoits 2011). Few studies explicitly explore the benefits of weaker ties (cf. Granovetter 1973; Erickson 2003); however, Thoits, in her 2011 review, draws on social support theory to suggest distinct provisions of primary and secondary ties. Primary ties are defined as being small in number, informal, intimate and enduring, while secondary ties tend to be greater in number, with more formal interactions guided by rules and hierarchical positions. Examples include ties formed through membership of voluntary and religious organisations which are frequently applied as measurements of social capital and social integration (e.g. Putnam 2000; Berkman et al. 2000). Thoits posits that an extensive secondary network increases the probability of finding a person with similar past experiences who can provide aspects of support such as information and advice, coping encouragement, and threat appraisal, that may be unavailable from strong ties. In the presence of direct experiential knowledge, the secondary social tie can offer empathy, but also certainty about the appropriateness of emotional reaction and coping behaviours. In addition, secondary ties are more likely to be role models that can be observed and emulated, which is to say that they are more relevant as a reference group for self-evaluation than primary ties and therefore more likely to yield self-esteem, self-worth and perceived control. Some empirical evidence bears this out. Study participants, for example, more often chose colleagues as points of comparison than close ties (Clark and Senik 2010). Weak ties also extend the range of social roles which are associated with the positive outcome and coping tool ‘sense of control’ where it is supposed that the effortful accomplishments associated with role obligations provide the efficacy and belief that problems can be overcome (Ross and Mirowsky 2013). Perceived control has been shown to entirely mediate the money-happiness relationship (Johnson and Krueger 2006) and to buffer the negative effect of low income (Lachman and Weaver 1998).

Furthermore, connections to organisations have also been linked to improved well-being. Berkman and Syme (1979), for example, show that church attendance and other voluntary group membership reduces mortality, an effect independent of marital status, friends and family and frequency of socialising. The social capital literature provides additional evidence for the benefits of organisation-based connections. In the USA, data suggest a positive effect of ‘civic connections’ on happiness (Putnam 2000). Benefits to SWB may also be indirect; for example, Stolle and Rochon (1998) show that mixing with diverse others in organisations builds trust and reciprocity; in turn social trust is shown to be linked to SWB (Helliwell and Wang 2010).

Drawing together these strands of theory and evidence, it is hypothesised that both strong and weak ties have important psychological benefits that will influence the money-happiness relationship. Further, the extended reference groups of those with weak ties and the sense of control and self-worth that result from social involvement with a wider base of ‘others’, will have a separable and additional effect to strong ties alone. The socially isolated will be the most vulnerable to financial circumstances in terms of the size of effect it will exert on happiness.

## Analysis Part 1: Patterns of Connectedness

The aim of this section is to develop a measurement schema based on strong and weak ties that will provide a suitable explanatory variable for the subsequent analysis of resilience in part 2. In exploring the effects of both tie types in combination, this analysis uses the concept of ‘social integration’ which is usually defined as the presence of both inward and outward-looking ties, where the need for close personal relationships is fulfilled but where there is also attachment to the wider community (Berkman et al. 2000). Social integration and its opposite, social isolation, are taken to be states of being rather than simply the ends of a scale of connectedness. Reflecting this, and because integration is defined in terms of two dimensions, a typological approach is taken. Typologies are not uncommon in the study of personal networks (e.g. Cattell 2001; Bellotti 2008; Li et al. 2008) and have the advantage that they suit the multi-faceted nature of connectedness and reflect a position in which connectedness is considered irreducible to a matter of more or less.

The latent connectedness variable is taken to be category-like rather than scale-like in its nature and latent class analysis is used to develop the typology. The core assumption of the model is that the social connections of people can be classified into a typological structure and that the manifest measures used in the analysis serve as imperfect indicators of a real, but unobserved, entity. A common cause relation (Borsboom et al. 2003) is assumed; that is to say that an individual’s pattern of connectedness causes the observed variation in the manifest variables. An advantage of categorical latent variables over continuous is that a single type of a latent categorical variable can be interpreted and labelled considering its position relative to the two dimensions thus simplifying what might otherwise be handled as interaction terms in regression models.

The treatment of the latent variable as categorical is preferable for further reasons, despite the scale-like nature of the two dimensions onto which the latent variable is mapped. First, it forces the consideration of cut-off points; for example it would be difficult to argue that connection to three organisations is qualitatively much different to two, while the difference between zero and one may be of more substantive relevance. This is what De Boeck et al. (2005) call an ‘abrupt difference’ which represents a discontinuity despite the linearity implied by the measurement scale. A further beneficial aspect of this approach is that the states of integration and isolation are explicitly modelled.

**Fig. 1:**
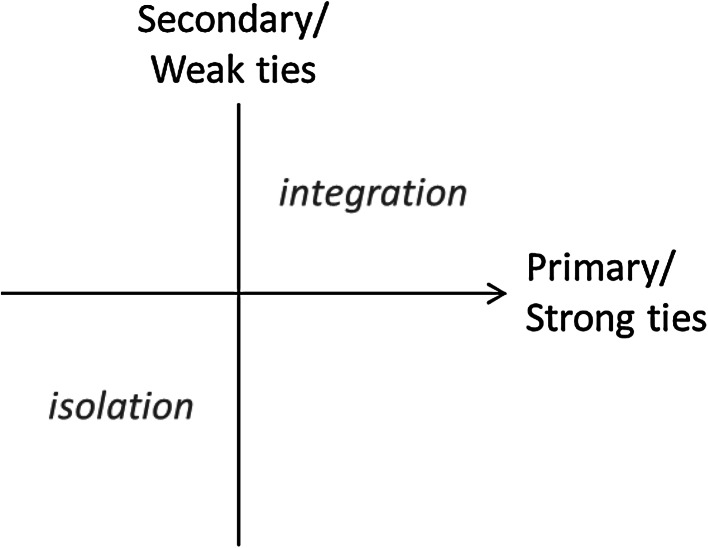
The four hypothetical spaces of connectedness based on strong and weak tie dimensions

As the types of connectedness have been identified a priori by the theory on strong and weak ties, the method applied is *confirmatory* LCA, meaning that the statistical outcomes are guided through a series of constraints. A simple framework is used to illustrate the approach and is shown in Fig. 1. It shows four ‘spaces’ of connectedness corresponding to the combination of being high or low on the two dimensions of strong or weak ties where those with both are socially integrated while those with neither are socially isolated. The ‘off-diagonal’ spaces containing individuals that have just one type of tie or the other serve as important points of comparison against which to compare the integrated and the isolated, allowing understanding of the contribution of each of dimension. This framework serves as the starting point for the development of the measurement model.

### Data and Model Specification

The British Household Panel Survey (BHPS) is used for the analysis. Not all relevant questions were asked to respondents in all years, thus variables from two waves (2003–2004) are combined into a single input file for the LCA model. Three dependent variables are used in the latent class analysis and they are described with a description of coding below.

#### The Strong Ties/Informal Connectedness Dimension

Two variables are used as indicators of strong ties. Firstly, the BHPS asks respondents to mention up to three friends with the question wording specifying that the mentioned friends can be family members but must be from outside the household. While a zero to three scale does not tell us much about total size of an informal network, it provides a critical distinction between those naming three, and those not; which is to say that an ‘abrupt difference’ is theorised to occur at the maximum value given the curtailed nature of the response options. 83 % mentioned three friends, 7 % mentioned two, 5 % had just one friend and 5 % could not name anyone outside the household.

Secondly, social support is an indicator of the quality of the relationships, i.e. the extent to which ties provide emotional support. Friends specialise in their role as companions for doing activities together, a function quite separate from emotional support (Bellotti 2008), and it is possible to have friends but still feel lonely, or alternatively to be perfectly happy with limited social contact (Cacioppo and Patrick 2008). The state of being isolated, may therefore be made up of two types of isolation; those feeling supported and those not. The model is set up to test this (Sect. 2.2) and confirms that the two types of isolation are empirically distinct. The addition of social support to the measurement model also has the advantage that its effect as causal mechanism is isolated.

The BHPS has a battery of five social support questions asking whether there is anyone available (1) who will listen (2) who will help in a crisis (3) to relax with (4) who really appreciates you and (5) you can count on to offer comfort. There were three possible answers of no-one, one person, or more than one person which are averaged into a single score.^1^ The resulting mean score is on a scale of 0–2, with higher scores representing a higher level of perceived support. The sample mean social support is 1.68, with a standard deviation of 0.43; the distribution is skewed to the left with almost half the sample having the maximum score of 2. As the questions were asked in the self-complete part of the questionnaire, respondents answering the survey by proxy or by phone are excluded from the analysis.

#### The Weak Ties/Formal Connectedness Dimension

Following the literature, ‘weak’ ties are indicated through activity with organisations (E.g. Stolle and Rochon 1998). The BHPS survey asks respondents whether they are members of, or are active in, a range of organisations including the following: political party, parents’ association, residents group, voluntary service group, community or social group, among others. Those claiming to be active, rather than simply members are selected for the analysis to avoid counting what have been called ‘check-book members’ where membership may not involve any social interaction (Putnam 2000). Additionally, the respondents are asked about the frequency of attending various activities including volunteering and community groups; those claiming to frequent these organisations weekly or monthly but had not claimed to be ‘active’ are recoded from 0 to 1 organisation. The number of organisations is coded as 0, 1, 2, and 3+; the higher counts being collapsed into a single category due to small frequencies.

### The LCA Model^2^

The model has two main aims. Firstly, in order to address the hypotheses, the aim is to have a solution that includes at least one class in each of the spaces of connectedness as it maps onto the dimensions of weak and strong ties. A second aim is to produce a model that fits the data well so that cases (individuals) can be assigned to classes with confidence, and those classes accurately represent the relationships of the indicator variables. A confirmatory latent class model is fitted in Mplus (Muthén and Muthén 1998) using the three dependent variables described above (friends, social support, organisations). Whilst the model is essentially confirmatory in nature, a further aim is to produce a model that fits the data well, and to those ends alternative models are also specified with more than four classes which are then evaluated for fit using the Bayesian Information Criteria (BIC). The BIC is based on the log likelihood but imposes a penalty based on the number of parameters in the model thus favouring more parsimonious solutions.^3^

Initially, a series of model constraints are added to ensure a class in each of the four hypothesised spaces of connectedness. The constraints are based on the assumed abrupt cut-off points at fewer than three friends (vs three) and having no organisational activity (vs any). Secondly, constraints are added that allow two types of isolation distinguished by the level of perceived support; this improves model fit. Finally, further exploratory models with 6, 7 and 8 classes are fitted and the statistics compared.

The final model is the six-class solution. The model with six classes produces a lower BIC than the 4 or 5-class solutions, while models with above 6 classes are either non-identified or show an increase in BIC.^4^ The 6-class model splits the integration space into two classes based on the level of organisational activity. The final solution has an entropy score of 0.97 which is an average of most likely class membership probabilities and can be interpreted as a measure of how well people were assigned to classes. This strong degree of certainty in class assignment reflects the confirmatory nature of the model and indicates that the classes are easily distinguished from each other.

### Results

A summary of the solution is shown in Table 1, which includes both the counts and probabilities of class membership and the means of the indicators.^5^ Class 1 contains 6.3 % of the sample and is made up of individuals who have restricted networks; each naming on average just 1.18 friends outside the household. People within this group are, on average, active with 1.32 organisations, and the mean level of social support is 1.56. Class 2 represents the most isolated of the typology and are the second smallest group at 5.3 % of the sample. As well as lacking emotional support (average 1.27) they are socially isolated naming on average 0.43 friends, and no organisations. In class 3 (5.0 %), despite having a restricted friend network (1.88 on average) and no organisational activity, the level of mean social support is moderate at 1.59. There are three classes where friend networks are unrestricted in size. Class 4 has the largest population with 43 % falling into this group; its members have high social support (1.68), but do not take part in organised social groups. Classes 5 and 6 both have high levels of perceived support as well as an unrestricted network of friends; they are distinguished only by the number of organisations in which they take part. People in class 5 (24.6 % of the sample) participate in just one organisation, whereas class 6 (15.8 %) contains people who are very involved, being active with an average of 2.28 organisations.

**Table 1:** Results of LCA model including indicator means and class sizes, based on BHPS data 2003–2004

Class label	Class 1	Class 2	Class 3	Class 4	Class 5	Class 6	Total
	Instrumental	Emotional isolation	Social isolation	Traditional	Integrated	Civic-minded	
Friends	1.18	0.43	1.88	3	3	2.98	
Organisations	1.32	0	0	0	1	2.28	
Social support	1.56	1.27	1.59	1.68	1.73	1.76	
N	892	754	715	6,102	3,492	2,241	14,196
%	6.3 %	5.3 %	5.0 %	43.0 %	24.6 %	15.8 %	100.0 %

For ease of description, as well as to make the model results more tangible, labels are assigned to classes. The labels are intended as convenient shorthand for the qualities of the class rather than a definitive statement about each individual it contains. Class 1 are “instrumental”. Although the motivation for joining organisations cannot be known from the indicator variables, an instrumental purpose for joining organisations is assumed given that members of this group have a restricted network of friends. Findings from previous research lend some weight to this assumption, for example, as a possible explanation for the surprising relationship between social skills and participation in activities among college students, Riggio et al. (1993) suggest that the reason for participating in organisations was in the *hope* of establishing supportive relationships. With this in mind, the label is assigned under the assumption that the organisational activity is a means to fulfil a social need.

Class 2 are “emotionally isolated”, while class 3 are “socially isolated”, a dichotomy demonstrated by Weiss’s research in the 1970s into a Boston organisation offering support to ‘parents without partners’. Many members of this group remained emotionally isolated even though they received comfort from the other group members. The wives of married couples who had moved to a new location, on the other hand, exemplified social isolation; despite the emotional support of their partners, the social isolation led to boredom and a sense of marginality (Weiss 1974). The two types of isolation, then, are theoretically and empirically distinct.

**Fig. 2:**
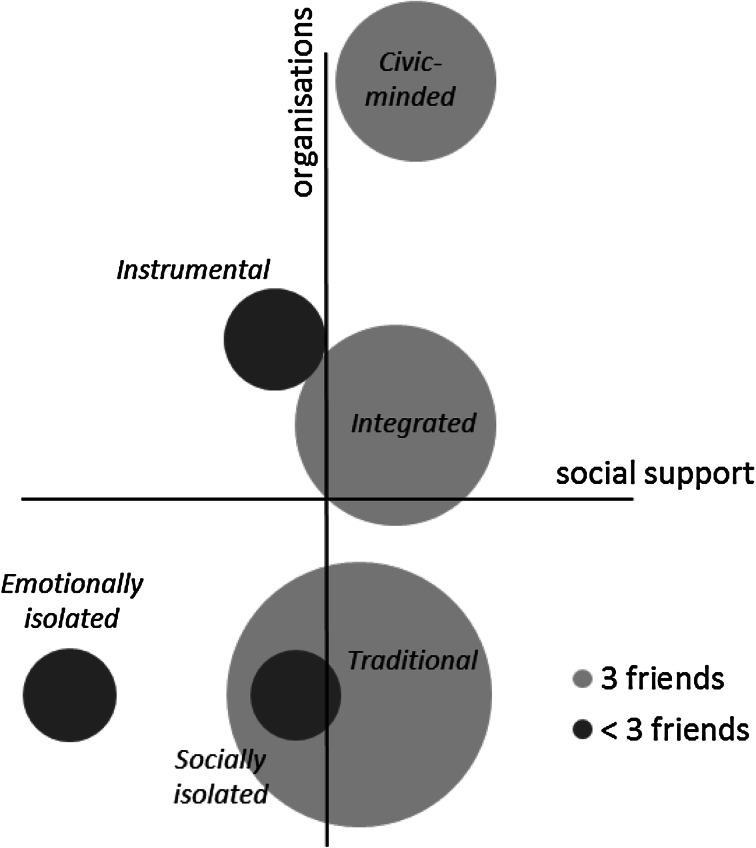
The latent classes in relation to the indicator axes of organisations and social support, *bubbles* are sized by the proportion of the sample in each class, and *shaded dark* if informal network is restricted to fewer than three friends

For class 4, the term “traditional” is assigned reflecting the label used by Cattell (2001) for networks of family, friends, and neighbours that are tight-knit in nature. Class 5 takes the label “integrated” based on Berkman et al’s (2000) definition as involvement with ties ranging from the intimate to the extended, combining two elements of the inward and the outward looking. I borrow Li et al’s (2008) “civic-minded” to describe the members of class 6 with their tendency to be very active in organisations. They are similar to, or rather a special case of, the “integrated” distinguished only by their high level of organisation-based connectedness. The position of the six classes is shown within the two-dimensional framework in Fig. 2.

It is hypothesised that the most resilient among the six classes will be the integrated and the civic-minded who have an unrestricted network of strong ties for social and practical support as well as a wide network of weaker ties to offer threat reappraisal and sense of control. As the civic-minded have a wider base of weak ties providing information on threat and coping, and more scope for gaining a sense of control, it is expected that they will be more resilient than the integrated. The traditional will do better than the isolated in terms of resilience because of the strong ties that bring self-esteem and support. The literature on the children of less nurturant parents suggests that the emotionally isolated will be the worst off in terms of resilience while the socially isolated may do better. In both classes, however, the small network will bring vulnerability in difficult economic times due to limits to the support available. As the instrumental group also miss out on the support of an unrestricted informal network, it is expected that they too will be less resilient than the civic-minded and the integrated.

It is expected that the life satisfaction of the socially isolated will be boosted to a greater degree by favourable financial circumstances compared to those with larger networks and that the integrated and civic-minded will be able to maintain higher SWB when experiencing financial stress. In accordance, the gaps in well-being by connectedness type will be smaller under good financial conditions but widen in stressful conditions.

### Stability of Connectedness

The LCA model is replicated using data from two later waves (2007–2008). The results are consistent with the earlier waves in terms of the nature (indicator means) and size of the classes. For those cases where indicator variables were non-missing at both times, the class assignment at the two time points are compared. This reveals that over half of the sample undergo a transition from one type of connectedness to another in the 4-year time span, perhaps reflecting changes in life course, job status, geographical mobility, or more generally the non-permanent nature of social ties, particularly to organisations. The changeable nature of connectedness has implications for the analytic strategy in the following section.

## Analysis Part 2: Connectedness as a Resilience Resource

### Data and Sample

The data used are from the last seven waves of the BHPS, from 2002 to 2008. The majority of variables are taken from the individual respondent modules, with additional information added from the household respondent module for the necessary measures to capture financial circumstances with greater accuracy. A purposeful sample is extracted for analysis which is made up of cases where connectedness, i.e. the LCA class assigned, is the same in both 2004 and 2008. The label ‘non-changers’ is given to this subsample; it contains 4,950 respondents which represents 45 % of the individuals with connectedness indicators present at both times. The data are converted into ‘long’ format with repeated measures within each individual denoted by ‘year’ which is numbered 1–7.

This analysis of the non-changers is intended to provide understanding of the link between connectedness and resilience to financial stressors, with results unclouded by the complexity of the dynamics of connectedness. By looking separately at this subsample, in effect the resilience resource is held as a time-invariant measure, allowing the relationship between financial circumstances and well-being through time to be explored in relation to the static resource. Among the changers, circumstances or events may influence both connectedness and personal finances, or indeed a change in connectedness may cause a change to the financial situation, and vice versa. In summary, the non-changers are analysed in isolation in part for analytic manageability, and in part for the clarity of the resulting conclusions.

The subsample of non-changers is broadly similar to the changers in terms of the socio-demographic and psychological profile. The non-changers are slightly older by just over a year on average, and have corresponding differences in economic status being slightly less likely to be a student or unemployed, and more likely to be retired. They are also score marginally lower in neuroticism. These factors are controlled for in the analysis.

### Variables

#### Outcome: Subjective Well-Being

The wave on wave correlations of life satisfaction are between 0.47 and 0.62 thus highlighting a degree of stability within individuals as would be predicted by set point theory, and stresses the need to specify between-person patterns of response in the analysis.

#### Explanatory Variable 1: Connectedness

For the main analysis, connectedness is treated as if it were an ‘observed’ variable, which is to say that the degree of certainty of class membership is not taken into account. It has been noted that this treatment of latent classification is problematic and possibly results in underestimation of standard errors (Bolck et al. 2004). However, the risk of bias is mitigated here; the possible impact of misclassification is minimal due to the high entropy score in the latent class analysis indicating that a good degree of accuracy is obtained in assigning cases to classes. Just 4.5 % of cases are assigned to a class based on a probability of membership of below 80 % and the potential bias of these is explored further in Sect. 3.5.

#### Explanatory Variable 2: Financial Circumstances

The effects of three different indicators of financial circumstances are included in the analysis, each reflecting a different aspect of income as it might influence SWB, as predicted by the literature. Household income is the most ‘objective’ in that it does not depend upon self-reporting but is constructed, using payslips where possible, by aggregating all sources of income including benefits, returns on investment, and so on, as well as earnings from employment. A number of transformations are applied to the income variable in order to increase comparability across households and time, and to correct for the non-normal distribution.

Firstly, income observations from subsequent waves are deflated to 2002 values to ensure comparability.^6^ The second conversion is the equivalisation of income across households which makes income directly comparable over households of different size and composition, and therefore across different years where changes in the household may have occurred. The conversion factor used is the McClements Equivalence Scale, a well-established scale based on the assumption that each additional person in the household raises costs. Thirdly, the distribution of household income is heavily skewed to the right, with a long tail representing a small number of households with very high incomes. To correct for this distribution the variable is transformed by taking the natural logarithm. Finally, this analysis follows others in assuming that observations at the very low end of the distribution of household income largely represent measurement error or under-reporting (Layard et al. 2008; Brewer 2013). To address this, the bottom percentile is excluded from the analysis, this meaning that 316 observations of under £390 per month are removed.

Subjective measures of financial situation have the benefit that the full circumstances can be taken into account with a single variable; it is likely that survey respondents consider savings and assets, rising or falling costs, family circumstances, job security and so forth, thus providing a more rounded understanding of personal circumstances than income alone. On the other hand, subjective measures are open to the same sources of bias as all subjective ratings (including life satisfaction) in that they will likely be influenced by personality, experience and aspirations, as well as current mood of the respondent. Conclusions are drawn from patterns of results from both the subjective and objective indicators of income thus mitigating the risk of biased results. The first of the subjective measures is a survey items which asks “How well would you say you yourself are managing financially these days?” with possible answers from the list: Living comfortably, Doing alright, Just about getting by, Finding it quite difficult, or Finding it very difficult. These are scored as 0–4 with ‘living comfortably’ as the highest score. The second is from a question directly following, where respondents are asked how their current situation compares with last year and have three options: better off, about the same, or worse off.

#### Controls

Several factors potentially confound connectedness, financial situation and life satisfaction that are controlled for in the analysis. These include employment status which influences household income and life satisfaction directly as well as providing opportunities for organisational activities (Li et al. 2003). Health has a strong relationship to SWB (Diener et al. 1999) and is controlled through ‘long-term ill’ as employment status. Social class is an additional factor that may influence happiness (Argyle 2001) and the relative constancy and security of certain occupations, even where badly paid, are considered economic resources (Goldthorpe 2006) which may influence resilience to economic shocks. Both higher life satisfaction and better social support are reported by those who are married or cohabiting (Argyle 2001), thus marital status is also controlled. Age is a further point of potential overlap between the key outcome and explanatory variables. To capture the relationship in full, a quadratic term of age is also included in the model, which is divided by 100 for reporting convenience. Being female is associated with reporting stronger affect both positive and negative (Diener et al. 1999) and is also included as a control.

The ‘big five’ personality traits are also controlled. Of the five, extraversion has been consistently associated with both SWB and connectedness while the opposite is true of neuroticism (Chan and Joseph 2000). The trait scores are constructed from a 15-question battery, each tapping into a perceived behaviour or internal state, with a resulting scale for each of 3–21. A final control for severe financial difficulty is also included. The following question, addressed to both mortgage payers and renters is used: “In the last 12 months would you say you have had any difficulties paying for your accommodation?” Like the other subjective indicators of income this control may be subject to bias, and further robustness checks are therefore used and described in Sect. 3.5. Descriptive statistics of the variables used in the analysis are shown in Table 2.

**Table 2:** Descriptive statistics of variables used in the analysis; categories omitted from the model are italicised

Categorical variables	%		%	Scale variables	Mean	SD
Worse off	22.5	*Professional*	16.3	Life satisfaction	5.3	1.2
Better off	23.0	Intermediate	51.7	Household income	7.5	0.8
Financial trouble	4.5	Semi-/routine	22.1	Financial situation	3.0	0.9
*Employed*	58.5	Never worked	9.9	Age	43.1	19.1
Full-time study	7.2	*Married/cohabit*	51.2	Agreeableness	16.3	3.0
Unemployed	3.0	Divorced/separated	9.9	Conscientiousness	15.7	3.3
Retired	20.6	Never married	7.0	Extraversion	13.5	3.5
Care of family/home	6.7	Widowed	31.9	Neuroticism	11.0	4.0
Long-term ill	4.0	Female	54.5	Openness	13.3	3.7

### The modelling Approach

#### Conceptual

The terms and conceptual clarity of *protective* *factor* models are borrowed from the resilience literature (e.g. Luthar et al. 2000). These models are defined by the inclusion of interaction terms that contain both the risk (the indicators of financial circumstances) and the protective factor (connectedness). Masten and Wright (2010) describe “[a] classic ‘protection factor’” as one which “shows stronger effects at higher levels of risk. In other words, the importance of the explanatory variable is greater when the risk is higher, suggesting a buffering or ameliorative influence” (p. 215), thus is a suitable approach for testing the hypothesis that well-being gaps will be wider at times of financial stress.

#### Statistical

A multilevel modelling framework is used in which the longitudinal data are taken to be hierarchical in nature with measurement observations (level 1) nested within individuals (level 2). Multilevel modelling of observations within individuals is used with the aim of demonstrating that financial circumstances that vary through time have a differential impact on SWB depending upon connectedness, while accounting for clustering of observations within the individual. The degree of similarity among the observations within-person, compared to between-person is made explicit through the residual variances and expressed through the Intraclass Correlation Coefficient (ICC).

Covariates are included that are time-varying (such as income, job status and marital status) and time-invariant. The main time-invariant, or person-level, predictor used in the analysis is connectedness, not based on its inherent invariability but due to the purposeful extraction of the subsample of respondents for whom connectedness did not change in the period 2002–2008.^7^ The 7 years selected for examination are assumed to be drawn from a random sample of 7-year periods. A linear variable ‘year’ is included to capture average change over time, and therefore age is kept as a fixed value (mean age across non-missing values) to avoid collinearity.

### Results

#### The Empty Model

The variance components or *empty* model (model 0, Table 3) provides the ICC statistic before any covariates are added. The outcome variable is life satisfaction, and the person ID is used as the level 2 identifier. This model uses all 33,232 observations with non-missing life satisfaction across 4,933 individuals. The residual variance at level 2 is 0.83, compared to the total of 1.52 indicating that 55 % of the variance in life satisfaction can be explained at the individual level. The empty model also provides the residual variances and ICC, against which subsequent models are compared. The Akaike’s Information Criterion (AIC) is also reported, which is a useful statistic for comparing nested models, with lower values indicating better fitting models.

**Table 3:** Multilevel models of life satisfaction; coefficients statistically significant at *p* < 0.05 are in bold

Fixed part of model	Model 0		Model 1a		Model 1b		Model 1c	
	b	SE	B	SE	b	SE	b	SE
Year			−**0.01**	**0.00**	0.00	0.00	0.00	0.00
Income			**0.05**	**0.01**	0.01	0.01	0.00	0.01
Financial situation					**0.23**	**0.01**	**0.23**	**0.01**
Better off					**0.04**	**0.01**	**0.04**	**0.01**
Worse off					−**0.10**	**0.01**	−**0.10**	**0.01**
Instrumental							−0.04	0.07
Emotional isolated							−**0.34**	**0.10**
Socially isolated							−**0.45**	**0.08**
Traditional							−**0.20**	**0.04**
Integrated							−0.05	0.04
Constant	**5.27**	**0.01**	**5.31**	**0.02**	**4.61**	**0.03**	**4.75**	**0.04**

Random effects	Var	SE	Var	SE	Var	SE	Var	SE
Random intercept: person	0.83	0.02	0.82	0.02	0.71	0.02	0.70	0.02
Occasion residuals	0.69	0.01	0.69	0.01	0.67	0.01	0.67	0.01
Intra-class correlation	0.55		0.55		0.52		0.51	
AIC	92,672.4		92,479.2		89,930.7		89,896.4	
N: observations/individuals	33,232/4,933		33,173/4,933		32,730/4,931		32,730/4,931	

#### Main Effects Models

Model 1a shows that household income has a positive effect on SWB (b = 0.05, SE = 0.01); the effect size suggests that an average income would buy around a third of a point of satisfaction. Once the subjective indicators are added (model 1b), the coefficient estimate drops to 0.01 and is no longer statistically significant. The subjective perception of financial situation has a strong effect on satisfaction (b = 0.23, SE = 0.01) while feeling better off has a smaller but independent effect (b = 0.04, SE = 0.01). Feeling worse off has a negative influence on satisfaction with an effect size (−0.10, SE = 0.01) around twice that of the opposite experience of feeling better off, a pattern consistent with loss aversion theory.

The dummy variables for connectedness type are introduced into model 1c. All five have a negative coefficient, which is to say lower life satisfaction, when compared to the reference category of the civic-minded, although the difference is not statistically significant in the case of the instrumental and the integrated. After controlling for financial matters, the isolated classes have the most severe deficit to their SWB; the emotionally isolated by 0.34 and the socially isolated by 0.45 when compared to the civic-minded. The traditional also experience satisfaction 0.2 lower. The addition of connectedness improves the model fit in terms of the AIC as do the subjective indicators compared to the single indicator of income. Model 1c sees a proportional reduction of error in the residual variances of 15 % at level 2 (using the empty model as the baseline), and 3 % at level 1. The ICC reduces from 55 to 51 % signifying that more variance has been explained at the individual level than the occasion level.

#### Moderating Effects or ‘Protective Factor’ Models

Interaction terms between connectedness types and the indicators of financial circumstances are computed and added to the multilevel models firstly for household income (Table 4). Wave-centred income is used to generate the interaction terms in model 2b so that the main effects of the connectedness dummies signify the differences at median income. The AIC is slightly larger than in model 2a indicating that the additional parameters of the interactions do not improve the fit of the model compared to without. Despite this limitation to the explanatory power of the model, the coefficients are significant and revealing.

**Table 4:** Protective factor models; coefficients significant at *p* < 0.05 are in bold and *p* < 0.1 italicised

Fixed part of model	Model 2a		Model 2b		Model 2c	
	b	SE	B	SE	b	SE
Year	−**0.01**	**0.00**	−**0.01**	**0.00**	−**0.01**	**0.00**
Income	**0.08**	**0.01**	−0.02	0.03	0.00	0.03
Instrumental	−0.08	0.08	−0.08	0.08	−0.04	0.07
Emotional isolated	−**0.41**	**0.11**	−**0.39**	**0.11**	−**0.28**	**0.12**
Socially isolated	−**0.50**	**0.09**	−**0.52**	**0.09**	−**0.35**	**0.08**
Traditional	−**0.25**	**0.04**	−**0.27**	**0.04**	−**0.09**	**0.04**
Integrated	−*0.08*	*0.04*	−**0.10**	**0.04**	0.01	0.04
Instrumental × inc			**0.21**	**0.08**	**0.23**	**0.08**
Emotionally isolated × inc			**0.28**	**0.12**	**0.27**	**0.14**
Socially isolated × inc			0.10	0.09	0.04	0.09
Traditional × inc			**0.14**	**0.04**	**0.09**	**0.04**
Integrated × inc			**0.09**	**0.04**	0.06	0.04
Age					−**0.03**	**0.00**
Age squared/100					**0.03**	**0.00**
Full time study					**0.18**	**0.05**
Unemployed					−**0.34**	**0.04**
Retired					*0.08*	*0.03*
Family carer					−*0.05*	*0.03*
Long term ill					−**0.49**	**0.04**
Divorce/separated					−**0.28**	**0.03**
Widowed					−**0.23**	**0.05**
Never married					−**0.14**	**0.03**
Live alone					−**0.11**	**0.03**
Intermediate					0.02	0.02
Routine					0.00	0.03
Never worked					0.01	0.03
Female					**0.09**	**0.03**
Agreeableness					**0.03**	**0.00**
Conscientiousness					**0.04**	**0.00**
Extraversion					**0.01**	**0.00**
Neuroticism					−**0.07**	**0.00**
Openness					0.00	0.00
Financial struggle					−**0.28**	**0.03**
Constant	**5.47**	**0.03**	**5.49**	**0.03**	**5.49**	**0.16**

Random effects	Variance	SE	Variance	SE	variance	SE
Random intercept: person	0.81	0.02	0.80	0.02	0.57	0.01
Occasion residuals	0.68	0.01	0.68	0.01	0.66	0.0
Intra-class correlation	0.54		0.54		0.46	
AIC	91,444.7		91,454.4		84,370.7	
N: observations/individuals	32,855/4,932		32,855/4,932		31,114/4,674	

The main effect of income, independent of the interaction terms therefore representing the effect for the civic- minded, is not substantive in size or statistically significant; the happiness of the civic-minded is therefore not driven by household income. The interaction terms are all positive coefficients, which is to say that household income is more important to satisfaction in all of the other five classes of connectedness, and the effect is statistically significant for all but the socially isolated. The effect size is notably large for the emotionally isolated (b = 0.28, SE = 0.12) which is far larger than the average effect of log income of 0.08 (model 2a). The effect is also sizeable for the instrumental (0.21) and a little smaller but nonetheless greater than average for the traditional (0.14) and the integrated (0.09).

Model 2c includes the socio-demographic and personality trait control variables. The modest reduction in effect size of the traditional, and the drop in significance of the effect of the integrated, make evident that socio-economic and psychological features partially explain the relationship between money and happiness for these connectedness groups. However, the controls are unable to completely ‘explain away’ the effects seen for the instrumental and the emotionally isolated, where the coefficient estimates remain similar in magnitude and significance is maintained.

The format is replicated for subjective financial situation in Table 5. Like household income, the interaction effects are positive indicating that all five connectedness types gain greater satisfaction from better perceived finances in comparison to the civic-minded. The effects are statistically significant for the socially isolated (b = 0.17, SE = 0.04), the traditional (b = 0.06, SE = 0.02) and the integrated (b = 0.05, SE = 0.01). The finding is weakened but does not disappear when controlling for psychological and socio-economic factors. Unlike with household income, the civic-minded are not unaffected by how they feel about their situation, but the effect is weaker than for other types of connectedness.

**Table 5:** Protective factor models for perceived financial situation; coefficients significant at *p* < 0.05 are in bold and *p* < 0.1 italicised

Fixed part of model	Model 3a		Model 3b		Model 3c	
	b	SE	B	SE	b	SE
Year	−**0.01**	**0.00**	−**0.01**	**0.00**	−**0.01**	**0.00**
Income	0.01	0.01	0.01	0.01	0.00	0.01
Financial situation	**0.25**	**0.01**	**0.20**	**0.02**	**0.18**	**0.02**
Instrumental	−**0.03**	**0.07**	−0.20	0.14	−0.11	0.14
Emotional isolated	−**0.31**	**0.10**	−**0.48**	**0.20**	−*0.38*	*0.22*
Socially isolated	−**0.43**	**0.08**	−**0.93**	**0.15**	−**0.71**	**0.15**
Traditional	−**0.18**	**0.04**	−**0.37**	**0.08**	−**0.20**	**0.07**
Integrated	−0.04	0.04	−**0.19**	**0.09**	−0.10	0.08
Instrumental × fisit			0.05	0.04	0.03	0.04
Emotionally isolated × fisit			0.05	0.06	0.05	0.07
Socially isolated × fisit			**0.17**	**0.04**	**0.14**	**0.04**
Traditional × fisit			**0.06**	**0.02**	**0.05**	**0.02**
Integrated × fisit			**0.05**	**0.02**	*0.04*	*0.02*
Controls	No		No		Yes	
Constant	**4.66**	**0.04**	**4.82**	**0.07**	**4.87**	**0.17**

Random effects	Variance	SE	Variance	SE	variance	SE
Random intercept: person	0.70	0.02	0.70	0.02	0.52	0.01
Occasion residuals	0.67	0.01	0.67	0.01	0.65	0.0
Intra-class correlation	0.51		0.51		0.44	
AIC	91,126.8		91,144.9		84,284.6	
N: observations/individuals	33,139/4,933		33,139/4,933		31,374/4,675	

The modelled effects of feeling “worse off” are shown in Table 6. Like household income and subjective financial situation, the cost of such a shock to life satisfaction differs across connectedness types. The instrumental (b = −0.21, SE = 0.08) and socially isolated (b = −0.26, SE = 0.09) have a negative interaction term indicating that their life satisfaction suffers more than that of the civic-minded when feeling worse off compared to last year. The interaction term for the emotionally isolated is positive, suggesting that, contrary to expectations, their satisfaction does not suffer when feeling worse off; this coefficient is of borderline significance. There is no difference in the reaction to feeling worse off for the traditional or the integrated in comparison to the civic-minded.

**Table 6:** Protective factor models for better and worse off; coefficients significant at *p* < 0.05 are in bold and *p* < 0.1 italicised

Fixed part of model	Model 4a		Model 4b		Model 4c	
	b	SE	B	SE	b	SE
Year	**0.00**	**0.00**	0.00	0.00	−**0.01**	**0.00**
Income	**0.03**	**0.01**	**0.03**	**0.01**	**0.02**	**0.01**
Worse off	−**0.22**	**0.01**	−**0.19**	**0.03**	−**0.17**	**0.03**
Better off	**0.09**	**0.01**	**0.06**	**0.03**	**0.08**	**0.03**
Instrumental	−0.09	0.07	−0.05	0.08	−0.02	0.07
Emotional isolated	−**1.14**	**0.15**	−**0.48**	**0.11**	−**0.38**	**0.12**
Socially isolated	−**0.45**	**0.06**	−**0.48**	**0.09**	−**0.33**	**0.08**
Traditional	−**0.27**	**0.04**	−**0.28**	**0.04**	−**0.10**	**0.04**
Integrated	−**0.09**	**0.04**	−*0.08*	*0.05*	0.02	0.04
Instrumental × worse			−**0.21**	**0.08**	−**0.17**	**0.08**
Emotionally isolated × worse			*0.26*	*0.13*	*0.27*	*0.15*
Socially isolated × worse			−**0.26**	**0.09**	−*0.16*	*0.09*
Traditional × worse			−0.02	0.04	−0.02	0.04
Integrated × worse			0.00	0.04	0.00	0.04
Instrumental × better			−0.08	0.08	−0.10	0.09
Emotionally isolated × better			0.09	0.15	0.06	0.17
Socially isolated × better			0.04	0.10	0.08	0.10
Traditional × better			*0.07*	*0.04*	0.05	0.04
Integrated × better			−0.03	0.04	−0.03	0.04
Controls	No		No		Yes	
Constant	**5.50**	**0.03**	**5.49**	**0.04**	**5.39**	**0.16**

Random effects	Variance	SE	Variance	SE	Variance	SE
Random intercept: person	0.80	0.02	0.79	0.02	0.56	0.01
Occasion residuals	0.69	0.01	0.68	0.01	0.65	0.01
Intra-class correlation	0.54		0.54		0.46	
AIC	92,426.4		91,754.7		84,672.06	
N: observations/individuals	33,036/4,932		33,063/4,932		31,303/4,675	

#### Plotting the Results

**Fig. 3:**
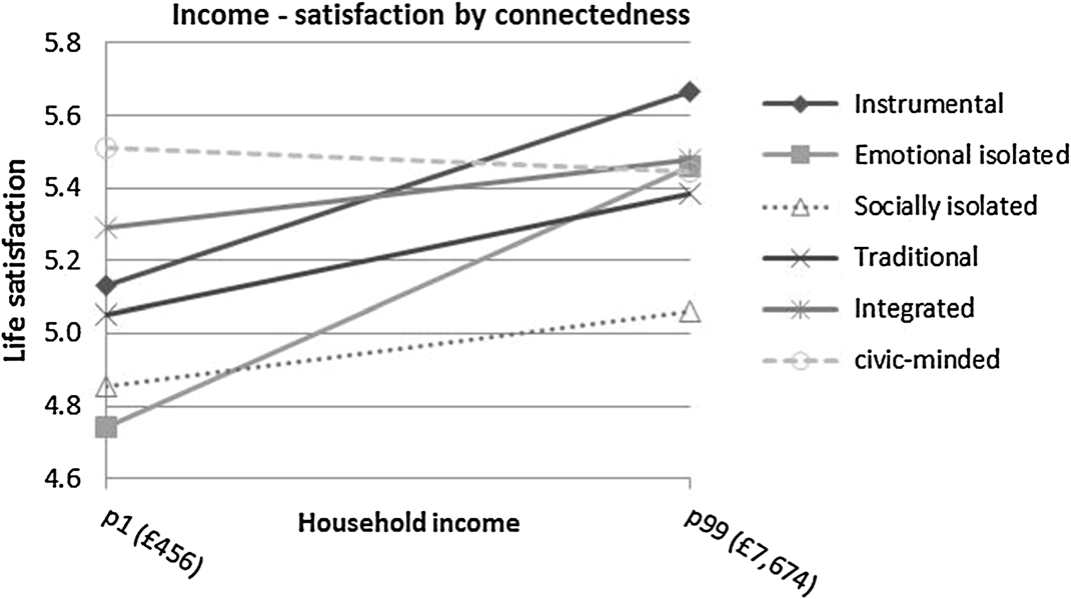
Plotted values of 1st and 99th percentiles of income on life satisfaction by connectedness, based on model 2b; non-significant coefficients in the model are shown with *dashed lines*

**Fig. 4:**
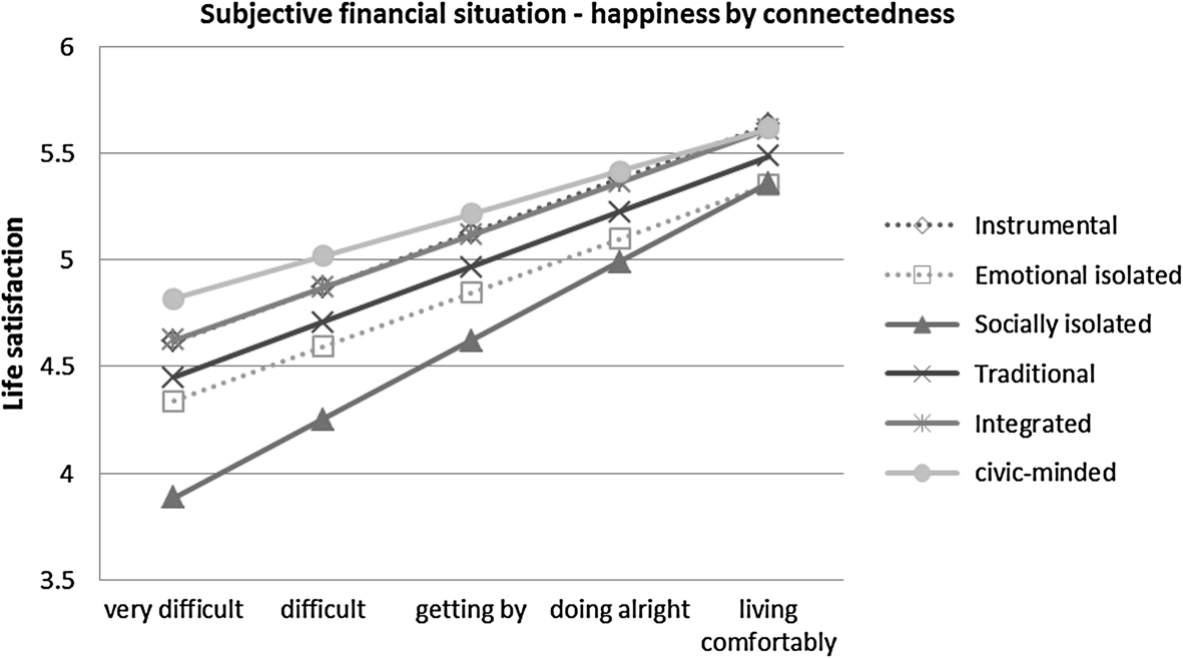
Plotted values of perceived financial situation on life satisfaction by connectedness, based on model 3b; non-significant coefficients in the model are shown with *dashed lines*

**Fig. 5:**
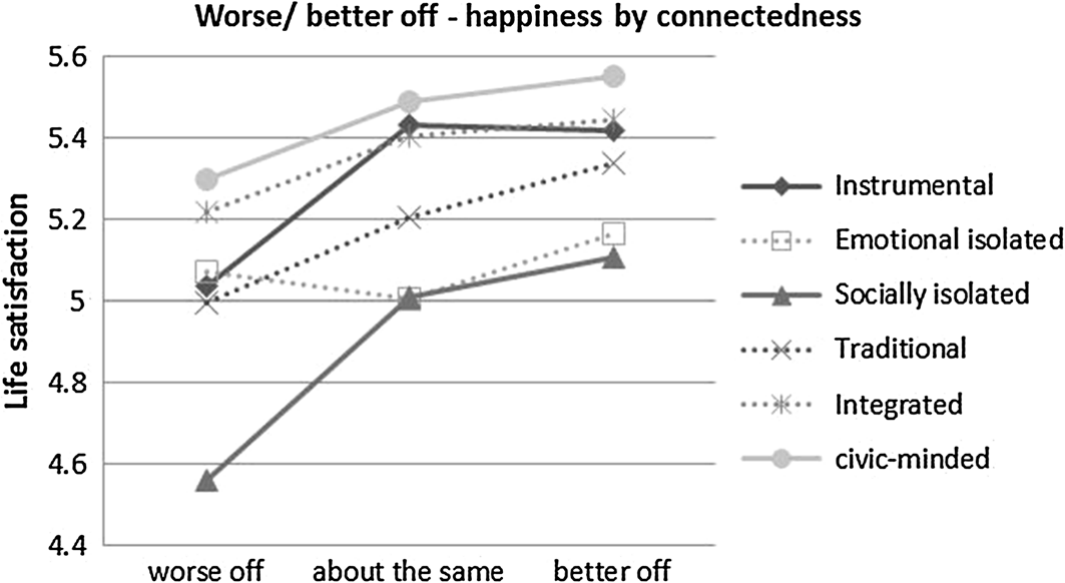
Plotted values of perceived worse, same, or better situation on life satisfaction by connectedness, based on model 4b; non-significant coefficients in the model are shown with *dashed lines*

With the aim of visualising the effect size, Figs. 3, 4 and 5 plot predicted values of life satisfaction for types of connectedness based on models 2b, 3b and 4b. The non-significant results are included but shown with dashed lines. Firstly, the 1st and 99th percentiles are used as predictor values of household income and the linear relationships by connectedness are shown in Fig. 3. The difference between the high and low ends of the scale therefore represents the entire range of possible outcomes. The two isolated classes have life satisfaction below five when under conditions of low income. The emotionally isolated, however, get more of a boost from income and by the 99th percentile (£7,674 per month) have increased satisfaction to 5.5 which is the same as the integrated and civic-minded at that level of income. Both the integrated and the civic-minded are able to maintain SWB at or above average when household income is low; the integrated at 5.3 and the civic-minded at 5.5. The steepest slopes are evident for the instrumental and emotionally isolated, while the lack of upward slope makes the civic-minded unique.

Figure 4 plots predicted values based on perceived financial situation with household income held at the median. The pattern of interactions of connectedness and perceived financial situation is more subtle; all have an upward slope i.e. feel more satisfied with life when perceiving a good financial situation. There is however, an overall pattern of ‘fanning in’ as we move up the scale to living comfortably, revealing that connectedness makes a bigger difference to SWB when under financial stress. The gap in satisfaction, for example, between the socially isolated and the civic-minded is around one full point when finding things very difficult, but the gap closes to around 0.25 when living comfortably, suggesting that money is bringing the status-related gains that are not provided by their social interactions.

This is echoed in Fig. 5 which uses the interactions with feeling better off and worse off where ‘about the same’ was the reference category; again the predictions are made with household income held at the median value. Following the pattern of the classic protection factor, the gap between the connectedness types is much larger in the presence of the stressor than not. The drop in life satisfaction experienced by the civic-minded (around 0.2) is around half of that experienced by the instrumental and the socially isolated (0.4) when financially worse off in comparison to last year.

### Robustness Checks

One possible alternative explanation for the large effects of income among the isolated is that the interactions do not reflect materialism, coping, or self-worth, but rather a greater need for income among these groups because they are less well-off to begin with. To eliminate need as a possible causal explanation of the results, model 2b is replicated but with observations in the lowest income quartile removed (online resource 1). The results are not weakened, in fact, to the contrary, the income-connectedness interaction terms are strengthened in size and significance suggesting that the effect of connectedness on the money-happiness relationship is more powerful above a cut-off at which we assume that basic needs are met. For example, the socially isolated now also require significantly more income for happiness (b = 0.44, SE = 0.15), compared to a non-significant effect shown in Table 4. The effect for the emotionally isolated increased from 0.28 (Table 4) to 0.66. For completeness, the models are also re-run with the top income quartile excluded to exclude the possibility that the result simply reflects that the civic-minded are better off. However, again, the results remain robust. Additionally, to ensure that the model results are not biased by the treatment of the latent variable as if it were observed, all models were re-run excluding the cases where latent class assignment was below 90 % certainty meaning around 1,000 observations are dropped (online resource 2). All results hold.

## Discussion

This analysis has shown heterogeneity, by connectedness type, in the degree to which money matters for happiness. In summary, money matters less to the satisfaction of the well-connected and more to the isolated. It is evident that connectedness has the power to narrow the well-being gap that exists between times of financial struggle and times of relative comfort. The purpose of the article has been to explore the effect of weak and strong ties in combination, and the best outcomes were seen for the civic-minded, a group with both strong supportive informal ties as well as extensive weak ties into the community. There is no relationship between household income and satisfaction for this group while subjective financial circumstances have a lesser effect than for others. Also confirming the hypotheses, we saw that the socially integrated are slightly less resilient and need a little more income to achieve the same degree of happiness and have equally good outcomes when feeling worse off compared to the year prior. The traditional also cope well with feeling worse off; they do, however, require larger incomes or better perceived circumstances to reach the same level of happiness as the civic-minded. It is apparent therefore that weak ties have a separable and additional benefit when in conjunction with high-quality supportive strong ties.

The emotionally isolated are the least resilient with respect to household income, but counter to expectations do not suffer greater costs to their well-being when feeling worse off, although the socially isolated do. The instrumental group are empirically distinct from the other types of connectedness in terms of their resilience patterns, which are more similar to the other isolated classes though with higher satisfaction overall. This confirms the role of strong supportive ties as a necessary condition, with the effect of partaking in organisations less effective without this solid base of friendship and support.

It was theorised that the patterns of resilience result, at least in part, from the psychological assets of self-worth, perceived control and the means of threat appraisal and so on that are gained through social interactions. This analysis suggests that the effect of organisations is as a method of social integration, a state that exists if the strong ties are present *in addition to* the weak. This research has important implications for future work in the field. Firstly, it is mistaken to assume that coping and resilience stem only from strong ties. Secondly, social status and the associated psychological benefits should be considered as central to understanding the money-happiness relationship.

Further areas of interest are highlighted by this analysis; the fact that the resilience of the civic-minded is far more evident in the objective measures that the subjective suggests that appraisals, not just satisfaction, may be influenced by connectedness. An additional implication relates to social comparison, which appears not to take place at all for the financially-struggling civic-minded despite their larger networks. Patterns of connectedness may therefore reduce the influence of social comparison on SWB, despite the increased opportunities for upward comparison.

### Limitations

A limitation of this analysis is that of the dual function of positive emotion, which influences both the outcome indicator (life satisfaction) whilst at the same time being a resilience resource (Fredrickson et al. 2003). While difficult to rule out positive affect or other dispositional factors entirely, the results of this analysis include examples suggesting that positive affect is not the underlying cause. The instrumental, for example, have life satisfaction very similar to (and not statistically different from) the civic-minded in the main effects model but find themselves functioning less well in the presence of a stressor. Similarly, the traditional have lower life satisfaction overall but deal relatively well with shocks. These cases, where different explanatory variables expose subtle differences in the processes, show that positive emotion as a resilience resource cannot fully explain away the observed effects. Future research possibilities include an examination of effects observed in resilience when *change* in connectedness occurs to provide evidence on the direction of causality and the role of dispositional factors.

## Conclusion

In the context of a literature that has largely neglected factors that intervene in the money-happiness relationship, this article highlights that money makes a much larger contribution to the happiness of the socially isolated than others, and conversely makes a much smaller contribution to the happiness of the socially integrated. The results are not driven by need, nor by the fact that the well-connected tend to be better off, but demonstrate that the benefits to life satisfaction stemming from money are psychological in nature, and that those psychological benefits can be alternatively experienced through specific patterns of social connectedness. The resilience to difficult financial circumstances that connectedness brings is not entirely explained by emotional support; there are observable separable benefits of weak ties when in conjunction with a foundation of numerous supportive strong ties. The outward-looking nature of the civic-minded and integrated patterns of social ties seems to reduce any effect of social comparison despite the increased size and reach of potential reference groups. In highlighting the role of connectedness as a resilience resource, the purpose of this research is not to claim that money doesn’t matter. No amount of self-worth or perceived control can be expected to override the basic needs which are met though material well-being. Rather, it points out that beyond a reasonable level that meets needs, those gaining satisfaction from money may do so through the enhancement of self-worth, self-esteem or control which can be provided by alternative non-material means such as connectedness.

## Electronic supplementary material

Below is the link to the electronic supplementary material.

Supplementary material 1 (PDF 184 kb)

Supplementary material 2 (PDF 305 kb)

## Footnotes

1 Before averaging, a multiple imputation procedure was applied to compensate for the possibility of respondents ignoring the questions they found harder to answer thus potentially elevating their mean score. The 5 items have a Cronbach’s alpha of 0.86 and so are suitable for treatment as a single scale.

2 As the indicators include continuous indicators, this model is more precisely labelled ‘Latent profile analysis’ but I use LCA as an umbrella term covering all models of this type.

3 The penalty imposed by the BIC is harsher than that of alternative fit statistics such as the AIC. The BIC better suits the purpose here, which is to develop an explanatory variable with the smallest number of categories necessary to test the hypotheses.

4 Model fit statistics are not discussed further due to the confirmatory nature of the model, and space constraints. However, tables are available on request from the author.

5 The variances are assumed to be constant across classes and are not shown.

6 The conversion rates are based on the retail price index (RPI). The RPI is calculated based on the costs of goods and services in a given period and is therefore a measure of inflation as it affects consumers. The index is calculated and released by the Office for National Statistics (ONS 2011).

7 This focus on cases where connectedness is stable means that fixed effects models are not a suitable analytic tool, there being no within-person variation on the explanatory variable of interest (Allison 2009).
