# Supplementary material for: For Whom Money Matters Less: Social Connectedness as a Resilience Resource in the UK
Source: Soc Indic Res. 2015 Jan 6;125(2):509–35. doi: 10.1007/s11205-014-0858-5 (PMC4703614; doi:10.1007/s11205-014-0858-5)
Supplement: Supplementary file 1 — Supplementary material 1 (PDF 184 kb) [file 11205_2014_858_MOESM1_ESM.pdf]

Online resource 1

Fixed part of model	1) Loginc > p25 b	se	2) Loginc > p25 & < p75 b	se
Year	**-0.01**	**0.00**	**-0.01**	**0.00**
Income	-0.07	0.04	-0.02	0.10
Instrumental	-0.13	0.08	-0.08	0.09
Emotional isolated	**-0.44**	**0.12**	**-0.35**	**0.13**
Socially isolated	**-0.63**	**0.09**	**-0.62**	**0.10**
Traditional	**-0.27**	**0.04**	**-0.24**	**0.05**
Integrated	**-0.12**	**0.05**	**-0.10**	**0.05**
Instrumental * inc	**0.30**	**0.13**	**0.47**	**0.22**
Emotionally isolated * inc	**0.66**	**0.20**	**0.99**	**0.35**
Socially isolated * inc	**0.44**	**0.15**	0.34	0.25
Traditional * inc	**0.17**	**0.05**	0.15	0.11
Integrated * inc	**0.14**	**0.06**	0.17	0.13
Constant	**5.50**	**0.04**	**5.50**	**0.04**
**Random effects**	**var**	**se**	**var**	**se**
Random intercept: person	0.75	0.02	0.80	0.02
Occasion residuals	0.59	0.01	0.63	0.01
**Intra-class correlation**	0.56		0.56	
**N: observations / individuals**	25009 / 4542		16631 / 3936	

Online resource 1 model 2b replicated with different samples, 1) excluding observations in the lowest income quartile and 2) with the highest quartile also excluded
