# Supplementary material for: For Whom Money Matters Less: Social Connectedness as a Resilience Resource in the UK
Source: Soc Indic Res. 2015 Jan 6;125(2):509–35. doi: 10.1007/s11205-014-0858-5 (PMC4703614; doi:10.1007/s11205-014-0858-5)
Supplement: Supplementary file 2 — Supplementary material 2 (PDF 305 kb) [file 11205_2014_858_MOESM2_ESM.pdf]

Online Resource 2

Model 2b

Fixed part of model	b	se
Year	**-0.01**	**0.00**
Income	-0.02	0.03
Instrumental	-0.08	0.08
Emotional isolated	**-0.39**	**0.11**
Socially isolated	**-0.52**	**0.09**
Traditional	**-0.26**	**0.04**
Integrated	**-0.10**	**0.04**
Instrumental * inc	**0.21**	**0.08**
Emotionally isolated * inc	**0.28**	**0.12**
Socially isolated * inc	0.10	0.09
Traditional * inc	**0.15**	**0.04**
Integrated * inc	**0.09**	**0.04**
Constant	**5.49**	**0.03**
**Random effects**	**var**	**se**
Random intercept: person	0.80	0.02
Occasion residuals	0.68	0.01
**Intra-class correlation**	0.54	
**N: observations / individuals**	32133 / 4789	

Model 3b

Fixed part of model	b	se
Year	**-0.01**	**0.00**
Income	0.01	0.01
Financial situation	**0.20**	**0.02**
Instrumental	-0.18	0.14
Emotional isolated	**-0.50**	**0.20**
Socially isolated	**-0.90**	**0.15**
Traditional	**-0.35**	**0.08**
Integrated	**-0.20**	**0.09**
Instrumental * fisit	0.05	0.04
Emotionally isolated * fisit	0.06	0.06
Socially isolated * fisit	**0.16**	**0.04**
Traditional * fisit	**0.06**	**0.02**
Integrated * fisit	**0.05**	**0.02**
Constant	**4.82**	**0.07**
**Random effects**		
Random intercept: person	0.70	0.02
Occasion residuals	0.67	0.01
**Intra-class correlation**	0.51	
**N: observations / individuals**	32103 / 4789	

Model 4b

Fixed part of model	b	se
Year	0.00	0.00
Income	**0.06**	**0.01**
Worse off	**0.07**	**0.03**
Better off	**-0.19**	**0.03**
Instrumental	-0.05	0.08
Emotional isolated	**-0.49**	**0.11**
Socially isolated	**-0.47**	**0.09**
Traditional	**-0.27**	**0.04**
Integrated	*-0.08*	*0.05*
Instrumental * worse	**-0.20**	**0.08**
Emotionally isolated * worse	*0.25*	*0.13*
Socially isolated * worse	**-0.23**	**0.09**
Traditional * worse	-0.03	0.04
Integrated * worse	0.00	0.04
Instrumental * better	-0.09	0.08
Emotionally isolated * better	0.10	0.15
Socially isolated * better	0.04	0.10
Traditional * better	*0.07*	*0.04*
Integrated * better	-0.03	0.04
Constant	**5.49**	**0.04**
**Random effects**	**var**	**se**
Random intercept: person	0.79	0.02
Occasion residuals	0.67	0.01
**Intra-class correlation**	0.54	
**N: observations / individuals**	32031 / 4788	

Online resource 2 model 2b, 3b and 4b replicated with cases excluded where latent class assignment was based on a probability of below 0.9
